# Interrelations of managing position with person-environment fit of gender-role orientation, and burnout

**DOI:** 10.1186/s12995-024-00403-y

**Published:** 2024-06-07

**Authors:** Eva Wacker, Axel Fischer, Julia Schorlemmer

**Affiliations:** 1 FOM Hochschule Für Oekonomie Und Management Berlin, Institut Für Gesundheit Und Soziales, Bismarckstr. 107, 10625 Berlin, Germany; 2 Charité – Universitätsmedizin Berlin, Campus Virchow-Klinikum, Augustenburger Platz 1, 13353 Berlin, Germany

**Keywords:** Person-Environment fit, Gender-Role Orientation, Burnout, Managerial position

## Abstract

**Background:**

In previous studies a moderator effect of management position could be found between Person-environment fit of masculinity, and burnout. Present study goals are to analyze previous fundings of the importance of the individual gender-role in relation to the work environment in more detail.

**Methods:**

In this cross sectional explanative study, an online survey took place using Gender Role Orientation Scale (GTS +) by Altstötter-Gleich and DearEmployee-Survey by Wiedemann et al. The sample consists of 891 participants–516 female (58%), 373 male (42%), among those 277 executives (32%) and 594 participants without managerial responsibility (68%), age 17–70 years (*M* = 29.86; *S* = 7.67). Four groups were divided according to P-E fit in femininity and P-E fit in masculinity, this enabled a more precise distinction between the participants. The proportions of executives were determined, and compared in each group by a ${\chi }^{2}$ -Test Hierarchical linear regression models predicting burnout and proving moderator effects of managerial position were calculated for each group.

**Results:**

The proportions of executives were the highest in the two groups with participants, who had a higher individual masculinity compared to their work environment. A moderator effect of managerial position between P-E fit in masculinity and burnout was found in group “Indifferent” (participants with lower feminity and masculinity compared with work environment). With a worse P-E fit in masculinity burnout values rise for individuals with no managerial position. On the other hand, among leaders burnout values decrease a worse P-E fit in masculinity.

**Conclusions:**

People with a high individual masculinity compared to work environment tend more to be selected as managers, regardless of the individual characteristics of femininity, which may generally lead to a highly masculine and less feminine leadership and corporate culture. This culture could increase burnout risk for people with low individual masculinity and high feminity scores compared to work environment as well as for persons with low individual masculinity and feminity compared to work environment, especially if they are not in a managerial position.

**Supplementary Information:**

The online version contains supplementary material available at 10.1186/s12995-024-00403-y.

## Background

In April 2022 European Agency for Safety and Health at Work published a Flash Eurobarometer with survey results about health and occupational safety in European countries. Among other, the participants were asked if they had experienced any health problems caused or made worse by their work. Most frequent answer was “overall fatigue” (37%) [1].

Microsoft published another survey in 2022, where in 11 countries around the world (Canada, US, Brazil, Germany, France, UK, Japan, Australia, New Zealand, China, India) in total 48% of employees and 53% of managers reveal that they are “already burnt out at work” (France: 49%/ 55%; Germany: 44%/ 54%; UK: 46%/ 49%) [2].

The prevalence of burnout has been increasing steadily in recent decades, and in the last decade there has also been a strong increase in the volume of sick leave days due to burnout—in 2012 to 2021, medical leave days due to burnout increased by more than 50% in Germany [3].

As research shows, women worldwide report higher levels of stress and burnout than men [1, 4, 5]. Burnout prevention as well as the question of why women report higher levels of burnout has become one of the important health issues of today.

As in the own previous studies, we doubt the meaningfulness of the methodological approach when comparing values of mental health between gender groups (as the participants are asked, which gender group they feel they belong to).

To begin with, this approach implies, that all women and all men live their gender in the same way, and there are no differences among women and among men. Second, this approach suggests that the given gender can only be associated with one role—that women have no masculine characteristics, and men have no feminine characteristics (gender role orientation describes feminine and masculine characteristics regardless of gender). Finally, it is also questionable which measures can be derived from such studies to prevent burnout. As studies show, women show in male dominated work teams higher burnout and stress values as well as take longer sick leave. Also men in teams dominated by women are on longer medical absence [6–9]. As only gender is taken into account as a group characteristic, a derived preventive measure could be to build teams with equal proportions of men and women. However, this is not realistic for all professions, and additionally it is known that feminity and masculinity can be developed regardless of gender. In summary, our goal is to test gender role orientation as a possibly better method than gender group comparison in predicting and preventing burnout.

### Person-environment fit

Person-environment (P-E) fit models compare the characteristics of the individual and work environment. It could be requirements at the workplace and resources of employees or a comparison of the expression of a specific characteristic in the working environment with characteristics of the individual assuming that a worse fit requires a higher adaptation and leads to more stress. The goal is to describe the effects of different P-E fit scores on health, well-being, subjective stress, work engagement of employees and similar [10, 11]. Some models speak of a correspondence [12, 13], other models use the expression “fit” [14, 15].

To determine a P-E fit score, the participants are usually asked to evaluate their environment in a special characteristic using a questionnaire, and to assess their own personality using the same questionnaire. A person-environment fit score is calculated by subtracting the individual value from the value of the environment. This person-environment fit score is then compared to various other variables, such as stress, well-being or burnout.

The subtraction results (P-E fit score) can have negative values (if individual characteristic score is higher than the characteristic score of the work environment) to positive values (if the individual expression of the respective characteristic is lower than that in the work environment). Values around zero represent the best person-environment fit. Naturally, this often leads to curvilinear relationships with other variables, requiring a transformation by squaring and taking the logarithm to achieve a linear relationship with other variables—since this is one of the requirements of Pearson's product-moment correlation and linear regression, which are frequently used to analyze the data [16]. This approach was also used in previous own studies [17, 18].

The weak point of this method is that through the transformation it is no longer recognizable whether the individual value is higher or the expression of the characteristic in the work environment. After the transformation, it is only visible how strong the difference or the P-E fit is. However, this can make a big difference in practice, which is why the procedure has already been criticized by other authors [16].

In order to make visible whether the individual value in femininity and masculinity is higher or the corresponding work environment score, the scores were ​​not transformed in the present study, but divided into four groups (see description below). Within the groups, the level of P-E fit and relations with other variables still can be analyzed.

### Gender-role orientation

The two dimensions of gender-role orientation feminity and masculinity describe the identification with gender stereotypes. In the present study Gender Typicity Scale [19] is used to indicate both feminity and masculinity. Feminity can be described as being connected with emotions–empathetic, sensitive, warmhearted–aside from tasks and goals. Masculinity might be characterized as goal and task orientation–confident, assertive, risk-taking, disciplined–autonomous from emotions and feelings [19]. Terms used synonymously in research are communion and agency [20] or expressivity and instrumentality [21], but in this study the terms femininity and masculinity are used consistently.

Studies showed that both constructs can be developed independently by an individual, regardless of biological sex. However, on average, higher femininity scores have been found in women and higher masculinity scores in men [6, 19].

By combining both feminity and masculinity four types are differentiated: feminine (high scores in feminity, low scores in masculinity), masculine (low scores in feminity, high scores in masculinity), undifferentiated or indifferent (low scores in feminity and masculinity), and androgynous (high scores in feminity and masculinity) [22, 23]. In the present study these types are used as orientation to build groups. Although, in this case not only the individual feminity and masculinity are used as reference, but P-E fit in feminity and masculinity – it is calculated by subtracting the individual score in feminity and masculinity from the corresponding value of work environment (see below).

Regarding mental health, well-being and resilience, research discusses in two directions. On the one hand, studies show that higher individual masculinity values ​​in particular are positively associated with higher values in mental health. This is referred to as masculinity model [Cook, 1985; Marsh & Byrne, 1996].

As an explanation, the authors speak of a *masculine bias* regarding the common image of mental health [Cook, 1985; Marsh & Byrne, 1996]. Mental health and well-being would correspond more to an image characterized by masculine characteristics such as a positive self-image, activity and high self-esteem than by feminine characteristics (being emotionally connected and authentically expressing positive and negative feelings).

A second model argues that individuals with both high femininity and high masculinity show the best scores on well-being, reporting better social skills and better adaptability [24–30]. This is known as the androgyny model [22, 23].

### Burnout

Burnout is a process towards an increasing emotional exhaustion and an increasingly cynical and distanced attitude towards one's own tasks and work (depersonalization). According to Job Demands-Resources (JD-R) model, mainly job demands lead to employees’ emotional exhaustion, while depersonalization is caused by subjectively lacking job resources [31, 32].

Women generally indicate higher burnout values [4, 5], additionally women remain twice as long in sick leave for burnout as men [3]. That goes along with the supposition, that women are more likely do emotional labor, as one dimension of burnout is emotional exhaustion.

Higher burnout values are often found in people of a younger age [33] and with longer working hours [34]

As in previous studies [17, 18], in the present work burnout is measured using the DearEmployee Survey [35] (see below).

### Managing position and burnout

Research does not show a uniform picture of the connection between leadership position and burnout. Some studies confirm managerial position as a protective factor towards development of burnout [36, 37]. Other investigations results reveal an opposite picture, burnout and stress levels of managers being higher than among employees [38–40]. There is also research, which could not find any link between managerial position and burnout [41].

As our own previous studies [17, 18] show a moderator effect of managerial position on the relationship between P-E fit in masculinity and burnout (for individuals with no managerial position there is a stronger relationship between a worse P-E fit in masculinity and higher burnout values), it should be analyzed in present work in more detail (see description below).

### Present study goals

Previous own studies have examined the relationship between the P-E fit in feminity and masculinity, burnout and work commitment [17, 18]. For methodological reasons, it was not specified whether the personal value in femininity and masculinity is higher or lower than the corresponding values ​​in the work environment, only the extent of the difference was calculated. Correlations of both gender and P-E fit in femininity and masculinity with burnout scores were demonstrated. Additionally, a moderator effect of managerial position in the correlation between P-E fit in masculinity and burnout was found.

In the current study, groups with different combination of high and low values ​​of the P-E fit in femininity and masculinity based on types according to androgyny model are to be distinguished from one another (depending on whether individual femininity and masculinity is higher or lower than that of the work environment) and the corresponding relationships are to be analyzed in more detail (see details below).

**Fig. 1:**
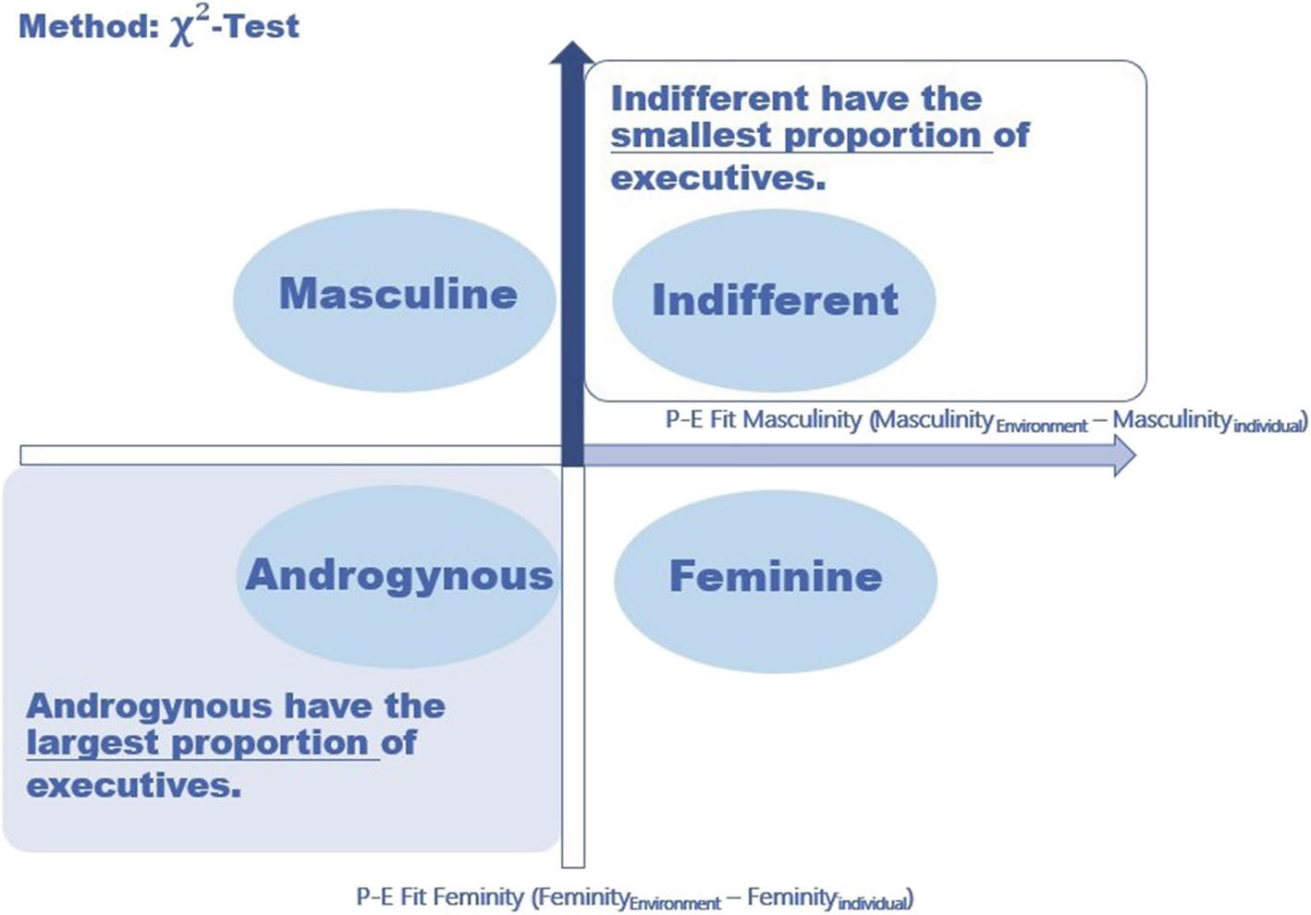
Expected results proving hypothesis H1 and H3 (Androgyny Model)

**Fig. 2:**
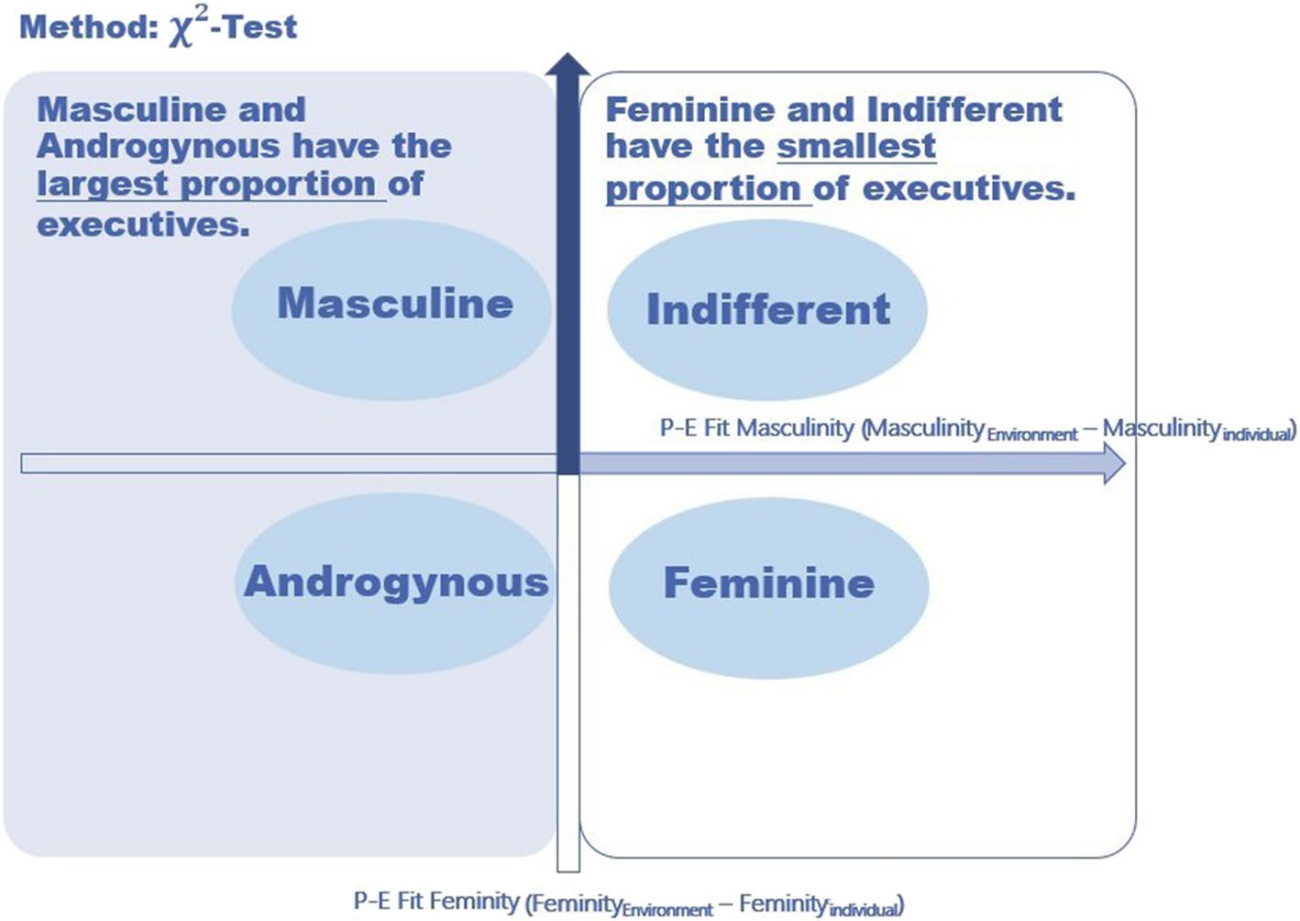
Results disproving hypothesis H1 and H3 (proving Masculinity Model)

Specifically, following the androgyny model it is being proofed, if the proportion of executives is the highest in the group with individuals indicating their feminity and masculinity higher than those of their work environment (group “Androgynous”), as their social skills and adaptability should be the highest between the groups (see Table 1, H1 and Fig. 1). Following the argumentation of masculinity model or masculine bias, the proportion of executives would be the highest in both groups with higher masculinity compared to the work environment – the group with higher masculinity and lower feminity (group “Masculine”), and the group with both higher masculinity and higher feminity (group “Androgynous”, see Fig. 2). An argument speaking for this is a work environment with high masculine and few feminine characteristics, in which leadership positions are given to people who are strongly masculine compared to work environment.

**Table 1:** Grouping according to P-E fit in feminity and masculinity and hypotheses

	P-E fit in Masculinity^b^ < 0	P-E fit in Masculinity^b^ ≥ 0
P-E fit in Feminity^a^ ≥ 0	Masculine	Indifferent H3: Group “Indifferent” has the smallest proportion of executives H4: In group “Indifferent” managerial position has the strongest moderator effects between P-E fit in masculinity and burnout
P-E fit in Feminity^a^ < 0	Androgynous H1: Group “Androgynous” has the largest proportion of executives H2: In group “Androgynous” managerial position has the weakest moderator effects between P-E fit in masculinity and burnout	Feminine

^a^P-E fit in feminity = feminity_workplace—_feminity_individual_

^b^P-E fit in masculinity = masculinity_workplace—_masculinity_individual_

**Fig. 3:**
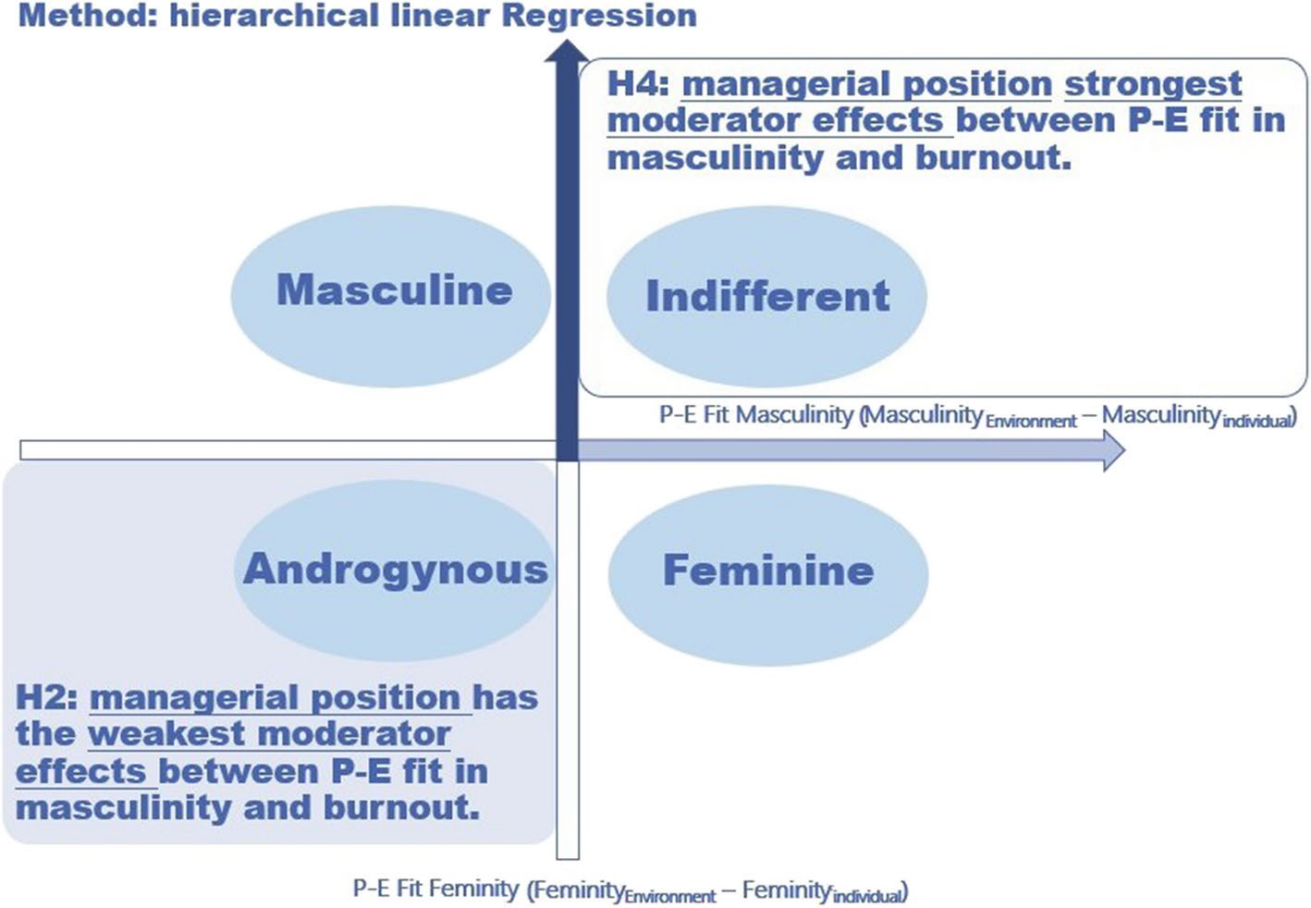
Expected results proving hypothesis H2 and H4 (Androgyny Model)

As next, the moderator effect of managerial position between P-E fit in masculinity and burnout discovered in previous studies [17, 18], should be analyzed in more detail. The moderator effect showed that burnout values increased with higher P-E fit in masculinity (worse fit), this relation was much stronger for people without managerial position. Following the androgyny model, in group with both higher masculinity and higher feminity compared to work environment (group “Androgynous”) this moderator effect should be weakest among all groups, as these individuals should have the best adaptability (see Table 1, H2 and Fig. 3). According to the masculinity model, the moderator effect should be the weakest in groups with high masculinity and low feminity values (group “Masculine”) as well as high masculinity and low feminity (group “Androgynous”) compared with work environment.

**Table 2:** Descriptive statistics of metrical variables

	*M (SD)*	*n*	1	2	3	4
1 P-E fit in feminity	-0.37 (0.57)	891	1			
2 P-E fit in masculinity	0.03 (0.80)	891	-.19***	1		
3 Age	29.86 (7.67)	870	-.02	-.12**	1	
4 Total working time	51.88 (10.20)	872	-.01	-.16***	.04	1
5 Burnout	2.88 (0.69)	891	-.19***	.06^a^	.001	.07*

^a^P-E fit in masculinity was transformed by squaring and taking a logarithm for a linear relationship with burnout

To calculate Pearson's *r*, outlier values were deleted and the linear relationship was approved

^*^p < .05

^**^p < .01

^***^p < .001

Following the expectations formulated so far, we assume the lowest proportion of leaders in the group with low values of masculinity and femininity compared to work environment (group “Indifferent”), as these individuals should have the worst adaptability according to androgyny model (see Table 1, H3 and Fig. 1).

**Table 3:** Executive proportions in all groups

	Counts Executives	Percentages Executives	Counts Non- Executives	Percentages Non- Executives	Total Counts	Percentages
Masculine	48	37%	83	63%	131	100%
Indifferent	30	23%	103	77%	133	100%
Feminine	91	26%	258	74%	349	100%
Androgynous	108	42%	150	58%	258	100%

From the perspective of masculinity model, the lowest proportion of leaders should be found in both groups with lower masculinity compared to the work environment – the group with lower masculinity and higher feminity (group “Feminine”), and the group with both lower masculinity and lower feminity (group “Indifferent”, see Fig. 2). This might be expected giving a work environment with high masculinity, where people who are strongly masculine compared to work environment would most likely and more often be promoted to managerial positions (masculine bias).

## Methods

Like in previous studies [17, 18], Gender Role Orientation Scale (GTS +) by Altstötter-Gleich [19] was applied to measure individual and work place feminity (8 items) and masculinity (8 items). The measured characteristics show to which extent a person identifies with traditional feminine or masculine stereotypes or how she perceives them in her environment. The questionnaire was validated with two samples (*n* = 1317 and *n* = 409), confirming the two-factor structure. Both scales show good reliability with McDonald's Omega 0.82 for expressivity and 0.84 for instrumentality. Checking the construct validity revealed among other interrelation between femininity and the construct ability to love (*r* = 0.58) measured by Trier personality questionnaire [42] as well as with Agreeableness *(r* = 0.43) according to NEO-FFI [43]. A good validity of the masculinity scale is confirmed by moderate correlations with the questionnaire on autonomy (*r* = 0.47) and self-esteem (*r* = 0.36) from the Trier personality questionnaire [19].

P-E fit in feminity was calculated by subtracting individual feminity ($\alpha$ = 0.79) from feminity of work environment ($\alpha$ = 0.83). The same way the values of P-E fit in masculinity resulted from deducting individual masculinity ($\alpha$ = 0.82) from masculinity of work environment ($\alpha$ = 0.88).

As in previous studies, burnout values were recorded using the DearEmployee Survey questionnaire (6 items, *α* = 0.86) [Wacker et al., 2021a, Wacker et al., 2021b]. The scale was validated with a random sample (*n* = 941) and showed good reliability with Cronbach's *α* = 0.84. Analysis of the validity showed, among other things, a high correlation with the COPSOQ burnout scale (*r* = 0.71) [Nübling et al.] and a strong connection with the number of acute complaints (*r* = 0.69) [Wiedemann et al.].

Participants were assigned to four groups, depending on their calculated values in P-E fit in feminity and masculinity. Group “Masculine” gathers individuals with a P-E fit in feminity higher than 0, and P-E in masculinity lower than 0–which means that their individual feminity was rated lower than work place feminity, and individual masculinity higher then work place masculinity (see Table 1). In group “Indifferent” are persons with a positive P-E fit in feminity as well a positive P-E fit in masculinity (individual feminity and masculinity was rated lower than work place feminity and masculinity). In group “Feminine” are participants with a higher individual feminity and lower individual masculinity compared to their workplace. In group “Androgynous” persons with a negative P-E fit in feminity and a negative P-E fit in masculinity are assigned, it means that the individual feminity and individual masculinity are both higher compared to their work environment (see Table 1).

### Study design, recruitment of participants

The sample for this study with an explanative cross section design was recruited over the intranet of a university specialized on working students with an already finished professional education. The link leading to the survey could be passed on to anyone. The online questionnaire took 5–7 min to fill out, survey participation was voluntary, without payment and could be canceled at any time. The sample size is *n* = 891, 516 female (58%), 373 male (42%) participants, among those 277 executives (32%) and 594 participants without managerial responsibility (68%), age 17–70 years (*M* = 29.86; *S* = 7.67).

### Statistical methods

Four hypotheses are to be proved (see also Table 1):

- H1: Group “Androgynous” has the largest proportion of executives.
- H2: In group “Androgynous” managerial position has the weakest moderator effects between P-E fit in masculinity and burnout.
- H3: Group “Indifferent” has the smallest proportion of executives.
- H4: In group “Indifferent” managerial position has the strongest moderator effects between P-E fit in masculinity and burnout.

Hypotheses H1 and H3 are proved with a ${\chi }^{2}$-Test. The a priori power analysis (*w* = 0.3, $\alpha$ = 0.05, *power* = 0.8) shows that a minimum of 88 persons is required. Hypotheses H2 and H4 are proved with a stepwise hierarchical linear regression with 11 predictors in all groups (4 steps, see Table 4). In Step1 the effects of gender and age are proved on burnout. In Step 2 Managerial position, number of employees, and total working time are added to the model as predictors. In the next step P-E fit masculinity and the interaction terms P-E fit masculinity*gender and P-E fit masculinity*managerial position were added. In the last step P-E fit feminity and the interaction terms P-E fit feminity *gender and P-E fit feminity *managerial position were added. The calculation of a minimum sample size results in *n* = 123.

**Table 4:** Hierarchical linear regression models predicting burnout

	Masculine		Indifferent		Feminine		Androgynous	
Predictor / Interaction term	$\Delta$ *R*^b^	β^a^	$\Delta$ *R*^*2*^	β^a^	$\Delta$ *R*^b^	β^a^	$\Delta$ *R*^b^	β^a^
**Step 1**	0.05*		.07*		.05***		0.01	
Gender^b^		-0.23*		-0.28**		-0.17**		-0.07
Age		-		-		-0.02		-0.04
**Step 2**	< 0.01		.01		.01		0.06**	
Managerial position^c^		-0.06		-		-0.04		-0.20**
Number of employees^d^		-		-		-		-
Total working time		-		-		0.05		0.06
**Step 3**	< 0.01		.05**		.04**		.01	
P-E fit masculinity		-		-		0.15**		-
P-E fit masculinity*gender		< 0.01		-		-		-
P-E fit masculinity* managerial position		-		0.24***		-		-0.09
**Step 4**	< 0.01		- < 0.01		.05***		.03*	
P-E fit feminity		0.03		-		-0.22***		-0.24
P-E fit feminity*gender		-		-		-		0.06
P-E fit feminity* managerial position		-		-		-		-
**Total *R***^b^	**0.06**		**0.11**		**0.14**		**0.11**	
Total *F*-Test	*F* (105) = 1.56 on 4*, p*** = **.19		*F* (122) = 7.51 on 2,*, p*** < **.001		*F* (303) = 8.33 on 6,*, p*** < **.001		*F* (189) = 2.82 on 8,*, p*** = **.006	
*n*	110		125		310		198	

^a^β in complete model

^b^Gender encoding: 1 = female, 2 = male

^c^managerial position encoding: 1 = yes, 2 = no

^d^Number of employees in managerial responsibility

^*^p < 05

^**^p < .01

^***^p < .001

The sample size is met for all statistical methods in use with one exception–hierarchical linear regression in group “Masculine” has a sample size of only 110. In this case, a power of 80% could not be secured, so there is a possibility that existing effects could not be discovered due to the small sample size. However, this didn’t play a role in the model analysis, as a linear relationship (requirement of linear regression) was not met for variables *age, number of employees, total working time, P-E fit in masculinity*, and interaction terms P-E fit in masculinity*managerial position, P-E fit in feminity*gender, *P-E fit in feminity* *managerial position – these had to be excluded from the model in Group “Masculine”. Other requirements of linear regression (residual analysis, homoscedasticity, absence of highly influential values, and multicollinearity) were confirmed. For a linear regression model with two predictors the power analyses shows a minimum sample size of 68 subjects, which is met in this group.

In group “Indifferent” two variables did not have a linear relationship with the outcome variable, and were excluded from the model – *total number of employees* and the interaction term P-E fit masculinity*gender. Because of multicollinearity further variables were excluded: *P-E fit masculinity*, interaction terms P-E fit feminity*gender and P-E fit feminity*managerial position.

Because with the non-significant predictor variables the regression model did not meet the requirement of homoscedasticity, these variables were removed from the model – *age, managerial position, total working time* and *P-E fit in feminity*. The remaining of linear regression (normal distribution of residuals, absence of highly influential values) were confirmed.

In group “Feminine” variable *number of employees* does not have a linear relationship with *burnout* as outcome, and was removed from the model. Because of multicollinearity all interaction terms P-E fit masculinity*gender, P-E fit masculinity*managerial position, P-E fit feminity*gender and P-E fit feminity*managerial position were also excluded. Other requirements of linear regression (normal distribution of residuals, homoscedasticity and absence of highly influential values) were confirmed.

In group “Androgynous” three variables had to be removed from the model because of curvilinear relationship to the outcome variable: *number of employees, P-E fit in masculinity* and the interaction term P-E fit masculinity*gender. To avoid multicollinearity, interaction term P-E fit feminity*managerial position was removed from the model.

## Results

The descriptive analysis revealed no significant relationship between the categorial variables *gender* and *managerial position* ((${\chi }^{2}$ (1) = 1.30, *p* < 0.255). The descriptive statistics of metrical variables are shown in Table 2. It shows that with a higher P-E fit in feminity (lower individual feminity compared to work environment) values of P-E fit in masculinity decrease (higher individual masculinity compared to work environment), and a higher P-E fit in feminity is related with lower burnout values.

On the other hand, higher values of P-E fit in masculinity (lower individual masculinity compared to work environment) are connected with lower age and lower total working time. And finally, longer total working hours are linked to higher burnout values.

To prove H1, the proportion of executives in group “Androgynous” was compared pairwise with proportion of executives in all groups. No significant difference could be proved between group “Androgynous” and group “Masculine” (${\chi }^{2}$ (1) = 0.99, *p* = 0.321), a small significant difference between group “Androgynous” and group “Indifferent” (${\chi }^{2}$ (1) = 14.32, *p* < 0.001, *w* = 0.19), the differences of proportions of executives could be proved small, but significant between group “Androgynous” and group “Feminine” (${\chi }^{2}$ (1) = 16.78, *p* < 0.001, *w* = 0.17). Accordingly, hypothesis H1 could not be confirmed. Group “Androgynous” does not have the largest proportion of executives. To prove, whether groups “Masculine” and “Androgynous” both have the largest proportion of executives, the proportion of executives in group “Masculine” was comparted pairwise with proportion of executives in all groups. A small significant difference could be proved between group “Masculine” and group “Indifferent” (${\chi }^{2}$ (1) = 6.29, *p* < 0.012, *w* = 1.15), also a small significant difference in proportion of leaders between group “Masculine” and group “Feminine” (${\chi }^{2}$ (1) = 5.17, *p* < 0.023, *w* = 0.10), and no significant difference between group “Masculine” and group “Androgynous” as shown above.

To sum up, groups “Masculine” and “Androgynous” both have the largest proportion of executives (for an overview see Table 3).

A hierarchical linear regression model was calculated to prove the moderator effect of managerial position between P-E fit masculinity and burnout as stated in H2 (see Table 4). No moderator effect could be proved in group “Masculine” ($\beta$ =—0.13, *p* = 0.710, *SE* = 0.27), group “Feminine” ($\beta$ = 0.46, *p* = 0.078, *SE* = 0.19), and group “Androgynous” ($\beta$ = -0.26, *p* = 0.272, *SE* = 0.23), so hypotheses H2 could not be confirmed. Additionally to the standardized coefficients their 95% confidence intervals are shown in Additional File 1, Table 1.

The weakest moderator effects of managerial position between P-E fit in masculinity and burnout are not in group “Androgynous”, no such moderator effects were found in all groups except group “Indifferent”.

To prove H3, the proportion of executives in group “Indifferent” is comparted pairwise with proportion of executives in all groups. A small significant difference could be proven between group “Indifferent” and group “Masculine” (${\chi }^{2}$ (1) = 6.29, *p* = 0.012, *w* = 0.15), but no significant difference between group “Indifferent” and group “Feminine” (${\chi }^{2}$ (1) = 0.63, *p* = 0.426). Small significant difference could be proved between group “Indifferent” and group “Androgynous” (${\chi }^{2}$ (1) = 14.32, *p* < 0.001, *w* = 0.19).

To prove, whether groups “Indifferent” and “Feminine” both have the largest proportion of executives, the proportion of executives in group “Feminine” was comparted pairwise with proportion of executives in all groups. A small significant difference could be proved between group “Feminine” and group “Masculine” (${\chi }^{2}$ (1) = 5.17, *p* < 0.023, *w* = 0.10), also a small significant difference in proportion of leaders was found between group “Feminine” and group “Androgynous” (${\chi }^{2}$ (1) = 16.78, *p* < 0.001, *w* = 0.16), and no significant difference between group “Feminine” and group “Indifferent” as shown above.

Accordingly, hypothesis H3 could not be confirmed. Group “Indifferent” with individuals having individual feminity and masculinity both lower than feminity and masculinity of work environment, does not have the smallest proportion of executives. Groups “Indifferent” and “Feminine” both have the smallest proportion of executives.

**Fig. 4:**
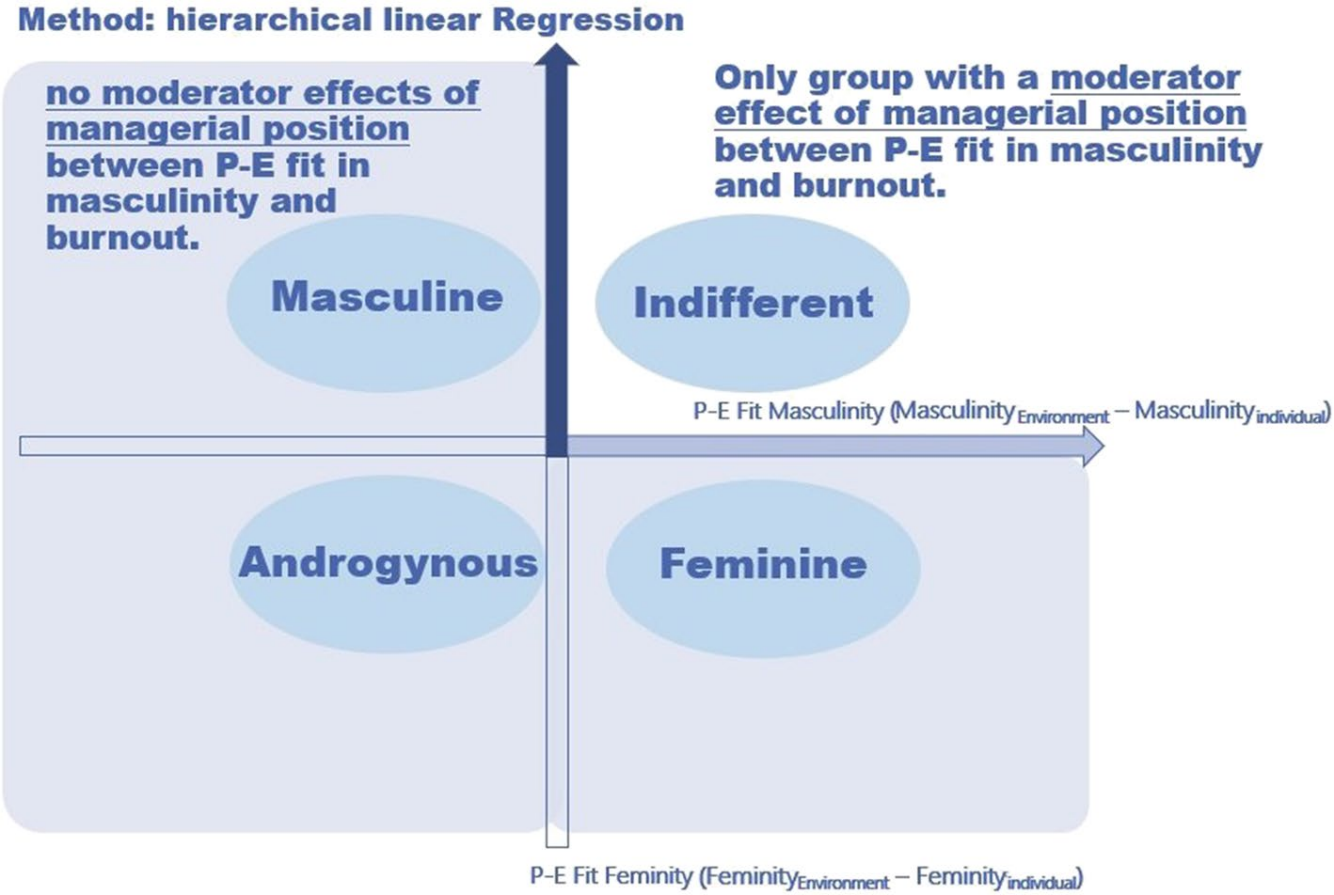
Results proving hypothesis H2 and H4

The H4 was tested by hierarchical linear regression as shown in Table 4. Group “Indifferent” is the only group in the sample with a significant moderator effect of managerial position between P-E fit in masculinity and burnout ($\beta$ = 1.72, *p* < 0.01, *SE* = 0.35). This way, H4 could be proved (see Fig. 4).

**Fig. 5:**
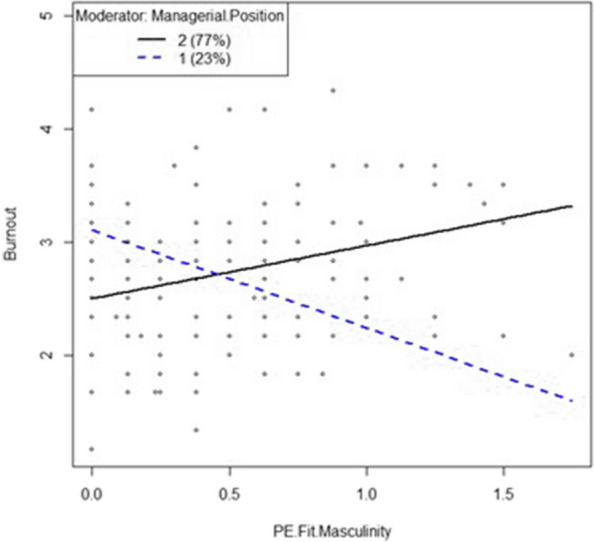
Moderator Effects of managerial position between P-E fit in masculinity and burnout. *Note*. Managerial Position Encoding: 1 = yes, 2 = no

The higher the P-E fit in masculinity (worse fit) for executives in group “Indifferent”, the lower burnout values (see Fig. 5). For individuals with no managerial position in this group the interaction is opponent to this–the higher the P-E fit in masculinity (worse fit), the higher their burnout values (see Fig. 5).

## Discussion

Hypotheses H1 “Group “Androgynous” has the largest proportion of executives” and Hypotheses H3 “Group “Indifferent” has the smallest proportion of executives” could both not be confirmed.

No significant difference in proportion of executives could be proved between group “Androgynous” and group “Masculine”– that’s both groups with a higher individual masculinity compared to masculinity of work environment.

As explained above, individuals with a higher individual masculinity than the work environment could be more likely to achieve a managerial position in a highly masculine work environment, as in a masculine company culture masculine characteristics are more connected with better social skills, strength, stability, reliability and adaptivity (e.g. positive self-image, assertiveness, discipline, goal orientation) as feminine characteristics (e.g. empathy, interest in authentic emotional well-being in self and others). This confirms previous study results that reinforce the masculine bias [5–7, 22]

Additionally, there were no significant differences in proportion of executives between group “Indifferent” and group “Feminine”–that’s both groups with a lower individual masculinity compared to masculinity of work environment. It seems that in a masculine work environment individuals with a lower individual masculinity could be less likely to achieve a managerial position.

The results of hypothesis testing of H1 and H3 show that despite of the proves of androgyny model, in practice, the masculinity model and *masculinity bias* dominate the selection of executives. Logically, this might lead to more leaders with high masculinity–some of them also having high feminity by chance (groups “Masculine” and “Androgynous”). In this way, a masculine leadership and a masculine company culture might be maintained and encouraged.

Hypotheses H2 “In Group “Androgynous” managerial position has the weakest moderator effects between P-E fit in masculinity and burnout” and H4 “In Group “Indifferent” managerial position has the strongest moderator effects between P-E fit in masculinity and burnout” both should examine closer the moderator effects of managerial position discovered in a previous studies [17, 18].

Surprisingly, the moderator effect of managerial position between P-E fit in masculinity and burnout could only be shown in group “Indifferent”–with individuals with as well lower individual feminity as masculinity compared with work environment. This way, H2 could not be confirmed, as the moderator effect did not occur in group “Androgynous”, nor in other groups except group “Indifferent”.

Hypothesis H4 could be confirmed, with a surprising relationship between P-E fit in masculinity and burnout among executives. For subjects with no managerial position the interrelation seems comprehensible–the worse P-E fit in masculinity, the higher burnout values. For executives an interesting correlation could be discovered: with lower individual values in masculinity compared with work environment (a worse P-E fit in masculinity) lower burnout values occur.

According to JD-R model, mainly job demands lead to employees’ burnout, buffered by job resources [32]. Thus, for persons in group “Indifferent” with lower individual masculinity for executives there could possibly be a subjectively lower demand to adapt to a more masculine work environment than for employees without managerial responsibility.

It is to be considered that managerial position has no direct effect on burnout in the model. So seemingly, managerial position only buffers the demand to adapt to a more masculine work environment, but is no general resource in burnout prevention. On the contrary, in group “Androgynous” persons with managerial position and lower employee responsibility indicate higher burnout values.

In this matter it is also interesting that in the other group with low values in masculinity compared to the work environment (group “Feminine”) managerial position does not show a similar moderator effect (see Table 4).

To sum up, in group “Indifferent” women show higher burnout values. For persons without managerial positions it became visible that with lower masculinity, burnout values rise. Being in a managerial position seems to work like a resource and take the pressure of having to adapt to a more masculine work environment.

### Study strengths and limitations

The presented work is a cross-sectional study that should be supplemented by longitudinal surveys and representative samples. In the present study a voluntary response sample is used, which is a non-probability sample, and could lead to self-selection bias. For instance, the sample is relatively young – leaving outlier values aside, the oldest participants are 37 years old.

Additionally, different approaches are possible when forming groups – for example, the mean distance from 0 for the P-E fit in femininity and masculinity might be calculated, thus forming nine instead of four groups (as we did following the androgyny model). Thus one group would gather persons with P-E fit values feminity and masculinity around zero (group with the best fit), and other groups with below average and above average P-E fit values in feminity and masculinity. According to a priori power analysis, this requires a significantly larger sample, since only a few people have extreme P-E fit values in femininity and masculinity. However, it could possibly expand the findings even further.

### Future research

An interesting fact is that in group “Masculine” none of the listed predictor variables contribute to the variance explanation of burnout, except *gender*.

In groups “Masculine” and “Androgynous” neither P-E fit in masculinity nor P-E fit in feminity explain burnout variance significantly. In both groups are subjects with a higher individual masculinity than the work environment. In group “Masculine” though, *gender* has an effect on burnout (men indicate lower burnout), however not in group “Androgynous”. The subject to investigate would be whether high femininity values can serve as a resource in burnout prevention to eliminate the gender effect. More specific the question would be first, if specific trainings could help to raise individual feminity values long term, and next to that – whether it effectively reduces psychosomatic complaints or burnout values.

In group “Indifferent” gender and the interaction between P-E fit masculinity and moderator variable managerial position could explain the most variance. In group “Feminine” P-E fit in masculinity and P-E fit in feminity both have a significant effect on burnout, which is not true for other groups. An explanation could be an assumed work environment with high masculine and low feminine characteristics, which are the complete opposite of the group individual characteristics, and both require an adjustment by the individuals in this group.

This might be a hint of possibly vulnerable groups when it comes to P-E fit in feminity and masculinity, which requires further research. Both groups (“Indifferent and “Feminine”) gather subjects with a lower individual masculinity compared to masculinity of work environment. Group “Indifferent” is a proportionally smaller group, group “Feminine” however gathers the most women and next to group “Androgynous” the largest proportion of men as shown in a former own study [44].

Future research could investigate first—if specific trainings could help to raise individual masculinity values long term, and second – whether it effectively reduces burnout values.

Finally, further research could prove, if specific leadership trainings could change the perception of work environment as more feminine (next to highly masculine characteristics) – and if this reduces stress and burnout values among employees.

## Conclusions

We can draw two important conclusions: 1) Managerial training should focus on masculine competencies, 2) when establishing a company culture feminine as well as masculine, values should be promoted.

Study results show that it is more likely to achieve a managerial position for individuals with a higher masculinity than the work environment, and less likely for individuals with a lower masculinity than the work environment. Therefore it seems reasonable to enforce masculine competencies like task- and goal orientation, and assertiveness by training to develop leaders.

On the other side, a highly masculine culture in a company could enforce burnout levels for individuals with a lower masculinity than their work environment. In case the individual feminity is lower than that of work environment (group “Indifferent”), burnout values of employees without a managerial position might increase with the subjective P-E fit in masculinity–the lower the individual difference compared work environment, the higher burnout scores. If the individual feminity is higher than the one of work environment, burnout scores grow with the subjective difference in P-E in feminity and P-E in masculinity. So for these groups it also seems helpful to train masculine competencies like setting boundaries, self-confidence as well as self- and time management as a burnout prevention in a highly masculine work environment.

Another important step might be to establish a stronger feminine company culture and feminine leadership as it is possible next to a masculine culture and leadership, as the biggest proportion of women and a big percentage of men [44] have a higher subjective individual feminity and lower individual masculinity compared to the ones of work environment.

To sum up, study results show that generally work environment is seen highly masculine by present sample, also persons with higher masculinity are preferred for leadership positions–regardless of individual feminity (groups “Masculine” and “Androgynous”). In this work environment two groups of people could be identified as vulnerable–group “Indifferent” (lower masculinity and feminity compared to work environment), especially people with no managerial position, as well as group “Feminine” (lower masculinity and higher feminity compared to work environment). Possible steps promoting and building a masculine as well as feminine corporate culture, among other things through training and selecting a masculine and feminine leadership–which would possibly lower stress and burnout values for many employees as well as strengthen their commitment to the company. Further burnout prevention measures could also be special trainings in masculine competencies for employees with low masculinity.

### Supplementary Information

**Additional file 1.** Data collected and analyzed in the study.

**Additional file 2.**

## Data Availability

Data generated and analyzed during this study are included in this published article and its supplementary information, Additional file 1.
