# Supplementary material for: Interrelations of managing position with person-environment fit of gender-role orientation, and burnout
Source: J Occup Med Toxicol. 2024 Jun 7;19:23. doi: 10.1186/s12995-024-00403-y (PMC11161922; doi:10.1186/s12995-024-00403-y)
Supplement: Supplementary file 1 — Additional file 1. Data collected and analyzed in the study. [file 12995_2024_403_MOESM1_ESM.pdf]

Additional File 1

Table 1 Hierarchical Linear Regression Models standardized Coefficients 95% Confidence Intervals.

	Masculine	Indifferent	Feminine	Androgynous
Predictor / Interaction term	β[1]<br>CI 95%	β[1]<br>CI 95%	β[1]<br>CI 95%	β[1]<br>CI 95%
**Step 1**				
Gender[2]	-0.23*<br>[-0.56,-0.05]	-0.28**<br>[-0.55, -0.10]	-0.17**<br>[-0.40,-0.09]	-0.07<br>[-0.32, 0.12]
Age	-	-	-0.02<br>[-0.02, 0.01]	-0.04<br>[-0.03,0.02]
**Step 2**				
Managerial position[3]	-0.06<br>[-0.34,0.18]	-	-0.04<br>[-0.23, 0.10]	-0.20**<br>[-0.46, 0.47]
Number of employees[4]	-	-	-	-
Total working time	-	-	0.05<br>[-0.01, 0.01]	0.06<br>[-0.01, 0.01]
**Step 3**				
P-E fit masculinity	-	-	0.15**<br>[0.05,0.36]	-
P-E fit masculinity*gender	< 0.01<br>[-0.15,0.15]	-	-	-
P-E fit masculinity* managerial position	-	0.24***<br>[0.04, 0.32]	-	-0.09<br>[-0.23, 0.05]
**Step 4**				
P-E fit feminity	0.03<br>[-0.40, 0.56]	-	-0.22***<br>[-0.50,-0.17]	-0.24<br>[-1.45. 0.62]
P-E fit feminity*gender	-	-	-	0.06<br>[-0.43, 0.58]
P-E fit feminity* managerial position	-	-	-	-

Note. * $p < .05$, ** $p < .01$, *** $p < .001$. [1] β in complete model; [2] Gender encoding: 1 = female, 2 = male; [3] managerial position encoding: 1 = yes, 2 = no; [4] Number of employees in managerial responsibility. CI = confidence interval.
